# A systematic review on risk factors for khat chewing among adolescents in the African continent and Arabian Peninsula

**DOI:** 10.1371/journal.pone.0263372

**Published:** 2022-02-03

**Authors:** Osman Abubakar Fiidow, Halimatus Sakdiah Minhat, Nor Afiah Mohd Zulkefli, Norliza Ahmad

**Affiliations:** Department of Community Health, Faculty of Medicine and Health Sciences, Universiti Putra Malaysia, Serdang, Selangor, Malaysia; LUMSA: Libera Universita Maria Santissima Assunta, ITALY

## Abstract

**Introduction:**

Khat is a flowering plant with stimulant effect on the nervous system and produce psychological dependence. Despite its harmful effects, the ingestion of khat has been part of cultural norms and the legality of khat varies by region.

**Objective:**

This systematic review aimed at critically evaluating the available evidence on the risk factors of khat chewing among adolescents.

**Methods:**

A systematic review was conducted on published research studies from five databases Scopus, PubMed, Science-direct, Ovid and google scholar using keywords khat chewing OR qat chewing AND associated factors OR risk factors OR contributing factors AND adolescents OR teenagers. Articles included were either cross-sectional, cohort, case-control or qualitative studies which were published between the year 1990 till present. Excluded articles were the non-English written articles, descriptive studies and irrelevant topics being studied.

**Results:**

Out of 2617 records identified and screened, six were included for the analysis and interpretation of the data. All included studies were cross-sectional study design. All six studies reported having family members who chewed khat significantly predict khat chewing among adolescents, followed by five articles for friends or peers who also chewed khat and four articles for male gender. Smoking was also found to have the highest odds (OR = 18.2; 95% CI: 12.95–25.72) for khat chewing among adolescents.

**Conclusion:**

The review highlights the crucial role of family members, friends or peers and male gender to predict khat chewing among adolescents. Effectiveness of health promotion programs to educate and reduce khat chewing among adolescents will require active participation of family members and friends.

## Introduction

Khat is also called *qat*, *jaad* or *qaad* is a flowering plant native to Ethiopia which produces effects analogous to those of amphetamine causing raising concerns about the health and social consequences. Khat chewing has been a common practice for many years in the Horn of Africa and the Arabian Peninsula where the khat plant is widely cultivated and known by a variety of names, including qaat and jaad in Somalia, and chat in Ethiopia [1] ((Al-Mugahed, 2008).

High prevalence of khat chewing has been reported in several countries, between 23.1% and 30.3% in Saudi Arabia [2] (Mahmoud et al., 2017) and from 6.95% to 64.9% in Oromia and Amhara regions respectively in the Ethiopia [3] (Astatkie et al., 2015), which was mainly related to spiritual [4] (Sikiru Lamina, 2010) and cultural [5] (Armstrong, 2008). Increasing intake among younger age groups as part of daily habit has also been reported [6] ((Mathewson et al., 2013). The beliefs that khat may enhance concentration [7] (Richard Hoffman & Mustafa al’Absi, 2011), performance motivation and strengthen their socialization, attract many adolescents of high schools and secondary school students to consume khat [8] (Meressa Kalayu et al., 2009).

The amphetamine-rich nature of khat leaves resulted in symptoms such as tachycardia, hyperthermia, dryness of the month, tachypnoea, mydriasis, and restlessness [9] (Dhaifalah & Santavý, 2004). Chronic ingestion of khat exposes the chewers to thrombocytosis, which may lead to myocardial infarction [10] (Al-Motarreb et al., 2002), ischemic heart disease, cardiogenic shock, arrhythmia [11] (Mega & Dabe, 2017), manic-like schizophrenia and distress secondary to withdrawal [12] (Alemu et al., 2020). Other reported adverse effects include erectile dysfunction [13] (Hassan, Gunaid & Murray Lyon, 2007), involvement in unsafe sex [14] (Lifson et al., 2017), psychotic experiences [15] (Odenwald & Khatkonsum, 2008), oesophageal cancer [16] (Bozzuto, Ruggieri & Molinari, 2010), low birth weight [17] (Khawaja Al-Nsour & Saad, 2008) among pregnant mother chewers, and lactation problem [18] (Glenice & Hagen, 2003) postnatally. Diverse risk factors have been observed across studies reflecting the cultural components to the risks of khat chewing among adolescents, with better understanding on the underlying risks needed in order for effective preventive and promotive intervention to be implemented. In view of its potential health, social and economic risks as well as the worrying prevalence among adolescents age group, this review is aimed to determine the risk factors of khat chewing among adolescents.

## Materials and methods

**Fig 1:**
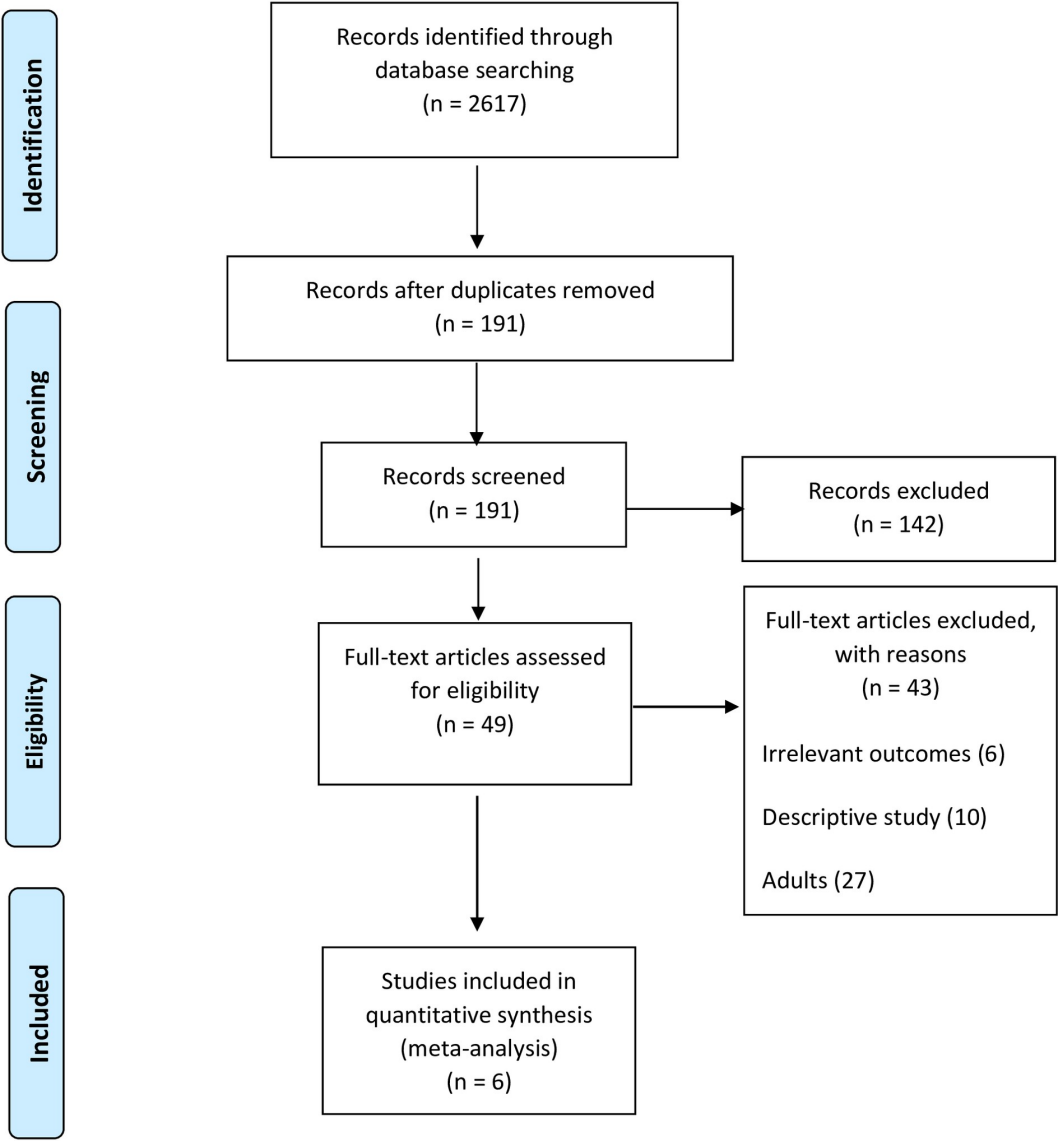
PRISMA flowchart.

The review was conducted and reported in accordance to the Preferred Reporting Items for Systematic Reviews and Meta-Analyses (PRISMA) statement (Fig 1) [19] (Moher et al., 2009).

### Eligibility criteria

Articles included were observational studies either cross-sectional, cohort or case-control studies and qualitative studies with publication period from the year 2010 to 2020. We excluded studies conducted among the non-adolescents age group, non-English language, descriptive studies, and protocols. Articles with irrelevant topics being studied and official reports from government agencies were also excluded. Khat chewing refers to the consumption of the leaves and stem tips of a flowery plant known as khat, which produced stimulating effect due to its amphetamine like effects [20] (Teferra, Hanlon & Jacobsson, 2011).

### Data sources and search strategy

We systematically searched for relevant articles published using five electronic databases from inception to June 2020, which included Scopus, PubMed, Science-direct, Ovid, and Google Scholar to retrieve studies of potential interest. The literature search was undertaken from five different databases using a combination of keywords of khat chewing OR qat chewing AND associated factors OR risk factors OR contributing factors AND adolescents OR teenagers.

### Study selection

Four researchers independently screened the titles and abstracts based on inclusion and exclusion criteria. Each study was recorded as include, exclude, or unclear. The full articles were retrieved for those recorded as include or unclear for further assessment, and the decision was made accordingly. Any discrepancies in the assessment were resolved by discussion leading to a consensus. According to the extensive basic and advance search of five databases with regards to the restricted inclusion and exclusion criteria, only these articles were found to be eligible to this study.

### Data extraction and quality assessment

Data extraction from all potential studies was documented in a table. The table included information on study design, study location, sample size, objective of the studies and major findings–risk factors associated with khat chewing among adolescents. Results selected to be included in the review had to possess specific measure estimates either calculated crude odds ratio, adjusted odds ratio, relative risk ratio, standardized beta coefficient with 95% confidence interval that does not include one or a p-value of less than 0.05 to have a significant factor included. Since the inclusion criteria of this study was to use multivariate.

The Crowe Critical Appraisal Tool (CCAT) was used to assess the methodological quality of the selected articles. The CCAT examined the study based on eight criteria which included preliminaries, introduction, design, sampling, data collection, ethical matters, result, discussion, and conclusion. The total score was then converted into percentage whereby the following categories were assigned to allow for comparison; poor quality (≤50%), acceptable quality (51–74%), high quality (≥75%) [21] (Crowe, Sheppard & Campbell, 2011).

## Results

### Study selection

A total of 2617 records were identified through five databases searching. After reviewing the titles and abstracts only 191 articles were included for further screening. Of those remaining, 142 articles were excluded due to non-English language, irrelevant outcomes, and study of substances other than khat. Forty-nine full text articles were screened, of which another 43 articles were excluded for the following reasons: non-adolescents study population, irrelevant outcomes, and descriptive studies. Finally, only six studies were included in this review [22–27] (Agili et al., 2018; Alsanosy, Mahfouz & Gaffar, 2013; Lakew et al., 2014; Kassa, Loha & Esaiyas, 2017; Mahfouz, Alsanosy & Gaffar, 2013; Reda et al., 2012), upon strict evaluation based on the predetermined eligibility criteria. Fig 1 shows PRISMA flowchart.

### Quality assessment

The Crowe Critical Appraisal Tool (CCAT) was used to assess the methodological quality of observational studies. The CCA tool consists of eight categories which is Preliminary, introduction, design, sampling, data collection, ethical matters, results, discussion, and conclusion. Each item has multiple items that simplify to score. Each category had scored 0–5 points. The total score was converted into percentage [28] (Akinla, Hagan & Atiomo, 2018). Quality assessments for each study was conducted using Crowe Critical Appraisal Tool. An established instrument used in assessing the quality of observational studies. Four studies were reported to be high quality while the other two were reported as acceptable quality as shown in Table 1.

**Table 1:** The Crowe Critical Appraisal Tool quality assessment.

Category		[25]	[23]	[22]	[27]	[24]	[26]
1.	Preliminary	4	4	4	4	3	4
2.	Introduction	3	4	3	4	4	4
3.	Design	3	4	3	4	4	4
4.	Sampling	3	5	4	4	3	5
5.	Data Collection	4	4	3	4	4	5
6.	Ethics	4	5	3	5	4	4
7.	Results	4	4	4	4	5	5
8.	Discussion	4	4	3	4	4	4
9.	Total score (/40)	29	34	27	33	31	35
10.	Percentage	73%	85%	68%	83%	78%	88%

### Characteristics of included studies

All articles included in this review were cross-sectional studies, with a total of 12157 participants were included in this study, dominated by high and secondary school students (Table 2). The studies were conducted between the year 2013 to 2018. Half of the included studies were conducted in Ethiopia while the other half were conducted in Saudi Arabia. All included articles employed probability sampling with randomization ensured during recruitment of respondents. Five out of six studies were only reporting the dependent and independent variables, except for a study by [25] (Kassa, Loha & Esaiyas, 2017) reported sex, living status during school age, and parental education level being the covariates involved in the study, with all included articles reported OR and 95% CI values.

**Table 2:** Factors associated with Khat chewing among adolescents.

No.	Authors/ year	Study objective	Methodology	Findings
1.	[23]	To investigate the prevalence of Khat chewing and related factors among intermediate and high school students of Jazan region.	**Study design** = Cross-sectional	Male, OR = 10.1 (95% CI: 4.88–17.38); Smoking, OR = 18.2 (95% CI: 12.95–25.72); Friend chewing khat, OR = 4.43 CI (2.78–7.37); Fathers chewing khat, OR = 1.68 CI (1.22–2.31); Brother chewing khat, OR = 2.65 CI (1.93–3.65)
			**Population and size** = 3764 intermediate or higher secondary school students during the academic year 2011/2012, aged13–21 years.	
			**Place =** Jazan Region Saudi Arabia	
			**Study Instrument =** A standardized questionnaire with pilot study of 160 students	
			**Analysis =** Epi Info and SPSSV 17 **Sampling =** three-stage cluster random sampling	
			Khat use measured using Lifetime and current use of Khat	
2.	[27]	To assess khat chewing among school adolescents in Harar town, eastern Ethiopia	**Study design =** Cross-sectional study design	Male gender, OR = 2.10 (95% CI: 1.50–2.93); Muslim religion, OR = 1.88 (95% CI: 1.17–3.04); Having friends who chewed khat, OR = 7.93 (95% CI 5.40–11.64); Availability of someone with a similar habit in the family, OR = 1.50 (95% CI: 1.07–2.11)
			**Population =** 1,890 secondary school students **Place =** Harar town Ethiopia	
			**Study instrument =** A self-administered structured questionnaire	
			**Analysis =** SPSS V15	
			**Sampling =** stratified sampling technique	
			Khat use measure using history of khat use	
3.	[24]	To establish the prevalence of khat chewing and associated factors among Ataye high school students and preparatory school students.	**Study design =** Cross-sectional	Male students, OR = 2.15 (95% CI: 1.02, 4.56); Peer chew Khat, OR = 3.14 (95% CI: 1.53, 6.41); Family chew Khat, OR = 2.68 (95% CI: 1.13, 6.37); Place of residency(urban), OR = 1.89 (95% CI: 1.0, 3.79)
			**Population and size =** 378 Ataye high school students	
			**Population =** Northern Shoa, Ethiopia	
			**Study instrument =** self-administered structured questionnaire	
			**Analysis** = Epi Info and SPSS V 17	
			**Sampling =** Multistage sampling	
			Khat use measured using Lifetime and current use of Khat	
4.	[26]	To reach reasonable estimate of the prevalence of khat chewing in the Jazan region and to investigate the different factors associated with this widespread habit.	**Study design =** observational cross-sectional	Smoking status, OR = 14.03 (95% CI; 10.76–18.30); Friend using khat, OR = 5.65 (95% CI; 3.92–8.14); Sister using khat, OR = 2.04 (95% CI; 1.11–3.74); Father using khat, OR = 1.45 (95% CI; 1.16–1.82); Brother using khat, OR = 1.56, 95% CI; 1.27–2.00)
			**Population and size =** 4,100 school students at the intermediate and secondary school	
			**Place =** Jazan Region SA	
			**Study instrument =** A standardized, self-administered questionnaire	
			**Analysis** = Epi Info and SPSS V 17	
			**Sampling =** three-stage cluster random sampling.	
			Khat use measured using Lifetime and current use of Khat	
5.	[25]	To establish prevalence of khat use, factors affecting students’ khat use, and also the effect of students’ khat use on their academic performance	**Study design =** cross-sectional in design	Smoking cigarette, AOR = 5.7 (95% CI: 2.3–14.3); Drinking alcohol, AOR = 3.0 (95% CI: 1.4–6.3); Having a family growing khat, AOR = 2.0 (95% CI: 1.1–2.5); Friend chewing khat, AOR = 3.1, (95% CI: 2.0–4.6); Male, AOR = 2.5, (95% CI: 1.6–3.8); Family Khat history, AOR = 2.0 (95% CI: 1.2–3.0); Ever practiced sex, AOR = 2.0 (95% CI: 1.3–3.0)
			**Population and size =** 1,655 students	
			**Place =** Sidama zone	
			**Study instrument =** self-administered questionnaire	
			**Analysis** = SPSS V 20	
			**Sampling =** Stratified sampling	
			Khat use measured using Lifetime and current use of Khat	
6.	[22]	To determine the prevalence and associated factors of Khat chewing among students of the high school in Jazan city, Saudi Arabia and to study its impact of on students’ academic performance.	**Study design =** Cross- sectional studies	Age 18 years, OR = 2.44, (95% CI: 1.12–5.33); Fathers use Khat, OR = 3.57, (95%CI: 1.98–6.46); Brothers use Khat, OR = 4.11, (95%CI: 2.28–17.64)
			**Population and size** = 370 high school	
			**Place =** Jazan City	
			**Study instrument =** self-administered questionnaire	
			**Analysis** = SPSS V 22	
			**Sampling =** simple random sampling	
			Khat use measured using Lifetime and current use of Khat	

### Risk factors of khat chewing among adolescents

Most of the studies included measured the lifetime and current use of khat (ever, current, and never) [22–26] (Agili et al., 2018; Alsanosy, Mahfouz & Gaffar, 2013; Lakew et al., 2014; Kassa, Loha & Esaiyas, 2017; Mahfouz, Alsanosy & Gaffar, 2013), with only one study measured history of khat use [27] (Reda et al., 2012). Age, gender, religion, grade, living with who, having family/friend/brother/sister/mother chewers, living with chewer, residential area, living status during school age, smoking cigarette, drinking alcohol, having family growing khat, participating in khat production and marketing activities, amount of monthly pocket money, friend smoking, friend use tobacco, received probation during last year, and feel depressed, parents’ educational level, family income, family attitude were identified risk factors with khat chewing among adolescents in all the articles reviewed.

However, having family members either sister, brothers or father consumed khat [23–27] (Alsanosy, Mahfouz & Gaffar, 2013; Lakew et al., 2014; Kassa, Loha & Esaiyas, 2017; Mahfouz, Alsanosy & Gaffar, 2013; Reda et al., 2012), friends or peers who are also chewed khat [22–27] (Agili et al., 2018; Alsanosy, Mahfouz & Gaffar, 2013; Lakew et al., 2014; Kassa, Loha & Esaiyas, 2017; Mahfouz, Alsanosy & Gaffar, 2013; Reda et al., 2012), male gender [23–25, 27] (Alsanosy, Mahfouz & Gaffar, 2013; Lakew et al., 2014; Kassa, Lpha & Esaiyas, 2017; Reda et al., 2012), and smoker [23, 25, 26] (Alsanosy, Mahfouz & Gaffar, 2013; Kassa, Loha & Esaiyas, 2017; Mahfouz, Alsanosy & Gaffar, 2013) were frequently reported risk factors significantly influenced the act of khat chewing among adolescents, with history of smoking had the highest odd of 18.2 (95% CI: 12.95–25.72) [23] (Alsanosy, Mahfouz & Gaffar, 2013). High odds were also observed for male gender, ranging from 2.1 (95% CI: 1.50–2.93) [26] (Mahfouz, Alsanosy & Gaffar, 2013) to 10.1 (95% CI: 4.88–17.38) [23] (Alsanosy, Mahfouz & Gaffar, 2013). The significant association between having family members chewing khat and khat intake among adolescents were reported in all six articles, with one study reported only a small number of parents disagree with the use of khat among their adolescent children [26] (Mahfouz, Alsanosy & Gaffar, 2013). Meanwhile, five articles reported significant association between having friends or peers chewing khat and khat chewing among adolescents [23–27] (Alsanosy, Mahfouz & Gaffar, 2013; Lakew et al., 2014; Kassa, Loha & Esaiyas, 2017; Mahfouz, Alsanosy & Gaffar, 2013; Reda et al., 2012), with four articles reported significant association between male gender and khat chewing among adolescents [23–25, 27] (Alsanosy, Mahfouz & Gaffar, 2013; Lakew et al., 2014; Kassa, Loha & Esaiyas, 2017; Reda et al., 2012), indicating the important roles of the both risk factors to influence khat chewing among adolescents. The non-significant association between peer influence and gender with khat consumption reported in the other articles reflecting he cultural role of khat chewing which is dominant in certain communities in these regions. On the other hand, being a smoker was reported as a significant risk factor in three articles [23, 25, 26] (Alsanosy, Mahfouz & Gaffar, 2013; Kassa, Loha & Esaiyas, 2017; Mahfouz, Alsanosy & Gaffar, 2013) reviewed. Most of the studies included measured the lifetime and current use of khat (ever, current, and never) [22–26] (Agili et al., 2018; Alsanosy, Mahfouz & Gaffar, 2013; Lakew et al., 2014; Kassa, Loha & Esaiyas, 2017; Mahfouz, Alsanosy & Gaffar, 2013), with only one study measured history of khat use [27] (Reda et al., 2012).

## Discussion

The review was conducted to identify the risk factors that significantly predict khat chewing among adolescents. Our review found, having family members and friends or peers who chewed khat, male gender and smoking frequently predicts khat chewing among adolescents, with the significant role of family members was reported in all six articles reviewed.

Numerous scholars reported khat as a malicious product that distracts the nation’s health, economy, and family dynamic as well as cultural or religious arguments [29] (Gudata, 2020). In certain society, khat consumption is accepted as an integral part of their culture, such as birth rituals, circumcision and marriage events. Additionally, among farmers, labourers and long-distance lorry drivers, chewing khat able to provide them energy for their labour-intensive daily activities, as well as for students when preparing for exams [30] (Laminal, 2010). Similarly, clan elders, and religious devotees were also frequently consumed khat for similar reason for all night sessions of prayer during Ramadan, which have been practiced over a long period of time [29] (Gudata, 2020) as cultural norms. Despite the reported scale of production and consumption, relatively little is known about the many ways in which khat interacts with lives, livelihoods, health, and economies, with attempts to legally prohibit khat use in some countries have been failed [30] (Laminal, 2010).

There has been substantial supporting evidence on the role of family members and peers in substance intake among adolescents, with peer and family use are direct correlates to use of substances by adolescents [31] (Tsering & Pal, 2009). Khat consumption is part of cultural norms in certain communities, which may also involve family interactions, parenting styles and practices, as well as family modelling and family backgrounds that can influence adolescents and youth behaviour [29] (Gudata, 2020). Its social and cultural norms was reflected by more than half of the female students and a quarter of the male students chewed khat with family members and relatives reported in one of the studies reviewed [29] (Gudata, 2020). Family and peer involve on both, the initiation and continuation of adolescent substance use, either due to poor parental behavior monitoring and control or acceptance towards the substance, manifested by their intake. Family particularly the parents serve as a role models and gatekeepers to both opportunities and barriers, for their children in imparting important health-related knowledge and appropriate behavior [32] (Al-Abed et al., 2014). Not only the vulnerability of adolescent age groups, but the social environment where the adolescent lives make him or her susceptible to use or disuse of various substances [31] (Tsering & Pal, 2009). The social acceptability towards khat chewing and socialization norms of this habit increase the likelihood of adolescents adopting the behavior [25] (Kassa, Loha & Esaiyas, 2017).

Khat chewing has been a common practice and traditionally accepted among the males and more culturally restricted in females [33] (Haile & Lakew, 2015), contribute to the lack of exposure among the females [34] (Gebrie et al., 2018). This significant difference may be due to the cultural acceptance of male practicing unusual things including khat chewing and other substances because khat generally considered “men’s thing” and viewed as unfavorable behavior for women [35] (Nakajima et al., 2013). The cultural taboo that excludes women for certain type of social [34] (Bebrie et al., 2018) activities may have contributed towards the role of gender as a risk factor for khat chewing [36] (Kassim, Rogers & Leach, 2014).

Majority of khat users also smoke tobacco but the temporal relationship or origin of this khat- tobacco use association is unknown. There has been argument on the similarity ways of consumption between khat and tobacco smoking [37] (Douglas & Hersi, 2010), and substance intake is frequently observed among men compared to women [38] (Becker, McClellan & Reed, 2017). Available evidence also showed that the habit of khat chewing reinforces the development of other habits such as cigarette smoking [39] (Birhane, 2014), as well as the effect of the khat consumption are enhanced mutually with cigarette smoking [12] (Alemu et al., 2020). The underlying psychobiological mechanisms of this link remain unclear, although it is possible that social cues and pharmacological priming associated with khat use may increase the likelihood and reinforcing effects of smoking [35] (Nakajima et al., 2013). Regional studies show that higher prevalence of cigarette smoking among khat and positive associations between levels of khat dependence and nicotine dependence [35] (Nakajima et al., 2013).

Many adverse effects have been linked with khat chewing which include impairment of mental [37–40] (Douglas & Hersi, 2010; Becker, McClellan & Reed, 2017; Birhane, 2014; Muluneh, 2018) and physical health such as myocardial infarction [10] (Al-Motarreb et al., 2002), ischemic heart disease [11] (Mega & Dabe, 2017), manic-like schizophrenia [12] (Alemu et al., 2020), erectile dysfunction [13] (Hassan, Gunaid & Murray Lyon, 2007), psychotic experiences [15] (Odenwald & Khatkonsum, 2008), oesophageal cancer [16] (Bozzuto, Ruggieri & Molinari, 2010), stroke, gastritis and hepatitis [40] (Muluneh, 2018). The urgent need to create better awareness on the potential negative consequences of khat chewing malpractice have been reported in several studies [23, 40–43] (Alsanosy, Mahfouz & Gaffar, 2013; Muluneh, 2018; Odenwald et al., 2007; Odenwald & Al’ansi, 2017; Wedegaertner et al., 2010).

To our knowledge, this is the first systematic review assessing the risk factors of khat chewing among adolescents. The use of five common databases as well involvement of four authors, ensured the strength of the search strategy and minimization of bias during selection of articles and data extraction. The review is also dominated by high quality articles. However, all articles reported the factors as risks towards khat consumption with none exploring the potential mediating and moderating role of potential factors such as gender, smoking or even family influence, which should be considered in future related research. The limitation of this study was only restricted to articles written in English; the studies were cross-sectional design in nature so it cannot show causality.

## Conclusions

The current review highlights the important risk of having family members and friends or peers who are also chewing khat, male gender and smoking to significantly predict khat chewing among adolescents. Health promotion program to educate adolescents on the harmful effects of khat chewing should target male adolescents and emphasize on the crucial participation of the family members and friends.

## Supporting information

S1 Checklist (DOC)
